# Interaction of Serum Copper and Neurometabolites on Executive Dysfunction in Unmedicated Patients With Major Depressive Disorder

**DOI:** 10.3389/fpsyt.2021.564375

**Published:** 2021-03-03

**Authors:** Xiaoxiao Liao, Shunkai Lai, Shuming Zhong, Ying Wang, Yiliang Zhang, Shiyi Shen, Hui Huang, Guanmao Chen, Feng Chen, Yanbin Jia

**Affiliations:** ^1^Department of Psychiatry, First Affiliated Hospital of Jinan University, Guangzhou, China; ^2^Jiangmen Central Hospital, Jiangmen, China; ^3^Medical Imaging Center, First Affiliated Hospital of Jinan University, Guangzhou, China; ^4^School of Management, Jinan University, Guangzhou, China

**Keywords:** major depressive disorder, copper, executive function, lenticular nucleus, proton magnetic resonance spectroscopy

## Abstract

**Objective:** The mechanism of executive function (EF) impairment in major depressive disorder (MDD) remains unclear. Previous studies have demonstrated that altered serum copper levels and neurometabolic alterations may be associated with the psychopathology and cognitive impairment of MDD. While, their inter-relationships in MDD remain uncertain. The present study aims to assess whether the interaction between serum copper levels and neurometabolic alterations is involved in the deficit of executive function (EF) in patients with unmedicated MDD.

**Methods:** Serum copper levels and EFs were measured in 41 MDD patients and 50 control subjects. EFs were evaluated by Trail Making Test, Part-B (TMT-B), Digit Symbol Substitution Test (DSST), Wisconsin Card Sorting Task (WCST), and Semantic Verbal Fluency testing (SVFT). Additionally, 41 patients and 41 healthy controls underwent proton magnetic resonance spectroscopy (^1^H-MRS) to obtain ratios of N-acetyl aspartate to creatine (NAA/Cr) and choline-containing compounds to creatine (Cho/Cr) in the lenticular nucleus (LN) of basal ganglia (BG). Finally, association and interaction analysis were conducted to investigate their inter-relationships.

**Results:** The results showed that patients performed worse in the DSST, WCST, TMT-B time and SVFT. Moreover, patients had higher serum copper levels, but lower NAA/Cr ratios in left LN of BG than healthy controls. In patients, serum copper levels were found to significantly negative associated with Categories Completed (CC) number of WCST (*r* = −0.408, *p* = 0.008), and positive associated with the Total Errors (TE) and Nonperseverative Errors (PE) number of WCST (*r* = 0.356, *p* = 0.023; *r* = −0.356, *p* = 0.022). In addition, the NAA/Cr ratios of left LN were found to significantly negative associated with VFS (*r* = −0.401, *p* = 0.009), as well as negative associated with serum copper levels (*r* = −0.365, *p* = 0.019). Finally, the interaction between copper and NAA may as influencing factors for SVFT and CC number of WCST in patients.

**Conclusion:** Our results indicated that the interaction of abnormal copper levels and NAA/Cr neurometabolic disruption of the LN may impact executive dysfunction, and this may relevant to the pathophysiology of executive impairment in MDD patients.

## Introduction

In China, the estimation of overall current, 12-month, and lifetime prevalence of Major depressive disorder (MDD) is 1.3–2.7%, 2.3%, and 2.8–3.3% (1). MDD is associated with cognitive cognitive deficits and it is established that depression increases the risk of cognitive impairment, particularly on executive functions (EF) such as memory, attention, and problem-solving (2, 3). Impairment of EF is widely recognized as a primary characteristic of MDD. Numerous studies have demonstrated that adults with MDD have mild to moderate EF deficits affecting inhibitory control, cognitive flexibility, and verbal fluency (4–6). Recent studies showed that 20–30% of individuals with MDD have pronounced executive dysfunction (7) persisting into the euthymic or more stable phases (3, 8). Furthermore, premorbid EF deficit is considered a trait-marker for adult MDD (9), and may prevent MDD patients from full remission (10). Nevertheless, the exact physiological mechanisms linking MDD with cognitive impairment are poorly known.

Trace elements play a role in the pathogenesis of MDD. Indeed, copper and zinc play important roles in MDD (11). Illustrating this relationship, Wilson's disease, or hepatolenticular degeneration, is a rare inherited autosomal recessive disorder of copper metabolism leading to copper accumulation in the liver and extrahepatic organs such as the lenticular nucleus and cornea (12). Among patients with Wilson's disease, up to 30 and 25% have symptoms of depression and cognitive impairment, respectively, and 25% patients are ultimately diagnosed with depression (13). Metabolic disturbance of copper is observed in patients with MDD, with higher serum copper levels (14). Inconsistently, previous studies examined the serum copper levels of patients with MDD and found lower or unchanged levels in patients with MDD (15). It is hypothesized that high serum copper is associated with higher oxidative stress, which plays physiological roles in cellular signaling and can cause cell damage and increased glutamate levels (16). Reactive oxygen species are associated with neurodegenerative processes and depression (17), and cognitive dysfunction (18, 19). While the underlying relationship between copper disturbance and executive function impairments in patients with MDD remain uncertain.

Imaging of patients with MDD reveals structural and functional abnormalities in the prefrontal cortex, anterior cingulate cortex, amygdala, hippocampus, basal ganglia and thalamus (20–22). As deep gray matter structures of the brain, the basal ganglia was critically implicated in the modulation of a wide range of motor, complex cognitive, and emotional processing as well as behavioral function (23, 24). Dysfunction of the lenticular nuclei, which are part of the basal ganglia, is observed in patients with MDD (25). Critical brain regions of adults with MDD house neurometabolic abnormalities that may reveal the biological mechanisms underlying the pathophysiology of their disorder (26, 27). Findings from neuropsychological and proton magnetic resonance spectroscopy (^1^H-MRS) studies support the important role of the basal ganglia in EF (28). Yet no significant differences in NAA/Cr or Cho/Cr ratios of the bilateral lenticular nucleus of the basal ganglia are evident in adults with MDD or in healthy controls (25). Likewise, a meta-analysis also finds no alteration of NAA in the basal ganglia of adults with MDD (29). Nevertheless, one study indicates that adults with MDD had a significantly lower NAA/Cr in the right caudate and a significantly higher mean Cho/Cr in the right putamen of basal ganglia (30). Higher choline and creatine concentrations are also found in the basal ganglia of adolescents with MDD (31). Indeed, basal ganglia have a role in disinhibition and planning and are partially involved in the regulation of emotion and cognitive functions.

Neurometabolic changes in critical brain regions are closely related to cognitive dysfunction in MDD. Lower NAA in the prefrontal cortex positively correlates with memory impairment in both adult and adolescent depression (25, 32). Altered NAA concentrations within the thalamus also parallel working memory impairment (33, 34). Yet an unchanged NAA/Cr ratio in the lenticular nucleus bears no relationship to working memory impairment related to MDD (25). Nonetheless, a lower NAA/Cr ratio in the left lenticular nucleus of the basal ganglia corresponds clearly to executive dysfunction in adults with bipolar II depression (28, 35). Furthermore, copper disturbance also have a close relationship with brain metabolic activity of the mood disorders. Previous studies have shown that increased copper levels were involved in the dysfunction of serotoninergic and dopaminergic metabolism in the LN of anxiety (36). Our recent study demonstrated that increased copper levels were negatively correlated with NAA/Cr ratios in the right LN of patients with MDD (37). But how copper disturbance interacts with the neurometabolic of the LN to result in cognitive impairment in patients with MDD begs investigation. Therefore, unique studies are needed to explore the interaction of abnormal copper levels and neurometabolic disruption may impact EF impairment in patients with MDD.

In consideration of this knowledge gap, we conducted the current study, which utilizes 3.0 Tesla ^1^H-MRS and assessments of neuropsychological function to explore the underlying relationship between neurometabolic change, copper disturbance and executive function in unmedicated patients with MDD. The metabolites NAA, Cho, and Cr were detected *in vivo*, with NAA and Cho reported as a ratio to Cr, since Cr level is relatively stable within the brain. Executive function was assessed by the Wisconsin Card Sorting Task (WCST), Trail Making Test Part-B (TMT-B), and Digit Symbol Substitution Test (DSST). We sought to find the interrelationships between copper levels, executive function, and neurometabolic alterations of LN on MRS in unmedicated patients with MDD.

## Methods

### Study Design

The present study was a cross-sectional design. We recruited unmedicated patients with MDD, as well as a comparable number of volunteers participated as healthy controls to analysis the difference and interrelationship of neurometabolic ratios, copper levels and executive function.

### Participants

Patients with MDD were recruited from the psychiatry department of the First Affiliated Hospital of Jinan University (Guangzhou, China) between October 2014 and April 2017. The age of the participants was restricted to 17–55 years to minimize the interference of aging and vascular diseases. Two experienced psychiatrists diagnosed each participant using the Structured Clinical Interview for Diagnostic and Statistical Manual of Mental Disorders (4 Edition) (DSM-IV) criteria Patient Edition. All patients were with scores of >20 on the 24-item Hamilton Depression Rating Scale (HDRS), and the scores of Young Manic Rating Scale (YMRS) were <6. The exclusion criteria were: (1) presence of other psychiatric disorders (bipolar disorder, schizophrenic episode, or panic attack) and symptoms; (2) history of any psychotropic medication, psychotherapy, or electroconvulsive therapy; (3) history of neurological or organic brain disorder; (4) alcohol/substance abuse within 6 months before study entry; (5) any physical illness demonstrated by personal history, or clinical or laboratory examinations; or (6) first-degree relatives having a history of neurological or mental illness.

A comparable number of volunteers participated as healthy controls were recruited via local advertisements. They were carefully screened through a diagnostic interview and the Structured Clinical Interview for DSM-IV Non-patient Edition (SCID-NP) to rule out the presence of current or past psychiatric illness. Additional exclusion criteria for controls were any history of psychiatric illness in first-degree relatives, and current or past significant medical or neurological illness.

All participants were right-handed and were scanned within 48 h of initial contact. The study was approved by the Ethics Committee of the First Affiliated Hospital of Jinan University, China. All subjects signed a written informed consent form after a full written and verbal explanation of the study.

### Executive Function (EF) Assessment

In total, 41 patients with unmedicated MDD and 50 healthy controls underwent cognitive assessment by two trained graduated students who were blinded to the diagnosis of each participant. Executive function assessment consisted of three tasks: Trail Making Test, Part-B (TMT-B), Digit Symbol Substitution Test (DSST), Wisconsin Card Sorting Task (WCST), and Semantic Verbal Fluency testing (SVFT).

### Trace Element Testing

In total, 41 patients with unmedicated MDD and 41 healthy controls provided blood for trace element detection. A 5-mL fasting venous blood sample was obtained at 7:00–8:00 A.M. by routine venipuncture from all participants. Blood samples were sent to a centralized laboratory that performed analysis following standard procedures. All blood samples were centrifuged and serum samples were obtained. The serum samples were kept at −20°C. Atomic absorption spectroscopy (AAS) was used to determine the levels of serum copper.

### Image Acquisition

In total, 41 patients with unmedicated MDD and 41 healthy controls underwent MRI scanning. ^1^H-MRS was conducted under resting conditions. Both magnetic resonance imaging (MRI) and ^1^H-MRS were performed on a clinical 3.0 T GE Discovery MR system (General Electric, Milwaukee, WI, USA) with a conventional gradient system and a standard eight-channel head coil. The subjects were lying in the supine position and the nasion served as a landmark. Ear plugs and foam pads were used to reduce noise and minimize head motion. Routine axial T_1_-weighted fluid attenuation inversion recovery (T_1_Flair) [repetition time (TR) = 1,000 ms, echo-time (TE) = 144 ms] and fast spin echo T_2_-weighted MR images (TR = 3,500 ms, TE = 102 ms) were obtained to confirm the absence of any structural and signal abnormality of the brain.

**Figure 1:**
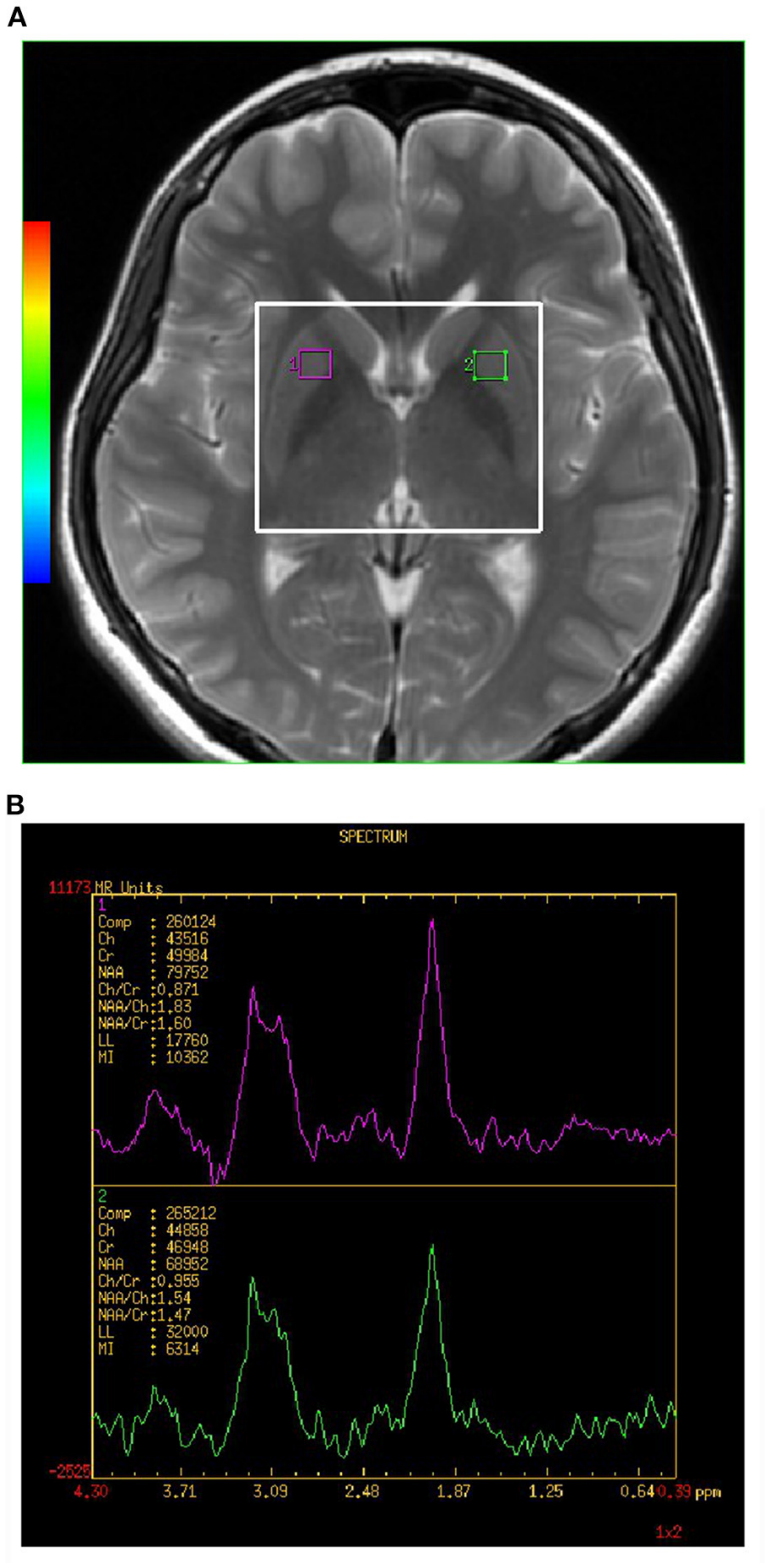
**(A,B)** Shows the VOI location in an MRI scan of the brain. Single section 2D multi-voxel 1H-MRS was acquired using a point resolved spectroscopy sequence (PRESS) with water suppression by a chemical shift selective saturation (CHESS) pulse. Magnetic resonance image (MRI) scan of healthy control subject showing location of magnetic resonance spectroscopy (MRS) of volume of interest (VOI) placed in the lenticular nucleus (LN) of basal ganglia (BG). The large white box represents the VOIx for MRS acquisition, and small white boxes depict the individual VOIs for spectral analysis. NAA, N-acetylaspartate; Cho, choline; mI, myo-Inositol; Cr, creatine.

All spectra were acquired using a 2D multi-voxel technique. Axial T_2_-weighted MR images were used for anatomic localization (TR = 3,500 ms, TE = 102 ms, slice thickness = 5 mm, without gap). For ^1^H-MRS, the positions of the volumes of interest (VOIs) were located in the bilateral LN showed in Figure 1A (large white box). The size of the VOI was 50 nominal voxels (7.5 × 7.5 × 10 mm). Single-section 2D multi-voxel ^1^H-MRS was acquired using a point resolved spectroscopy sequence (PRESS) with water suppression by a chemical shift selective saturation (CHESS) pulse. The acquisition parameters were: TR = 1,000 ms; TE = 144 ms; numbers of excitation = 1; spatial matrix = 256 × 256; field of view = 180 × 180 mm; slice thickness = 10 mm; and nominal voxel size of 7.5 × 7.5 × 10 mm. Additional saturation bands were placed outside the VOI to minimize lipid contamination from the scalp. Automatic prescanning was performed before each spectroscopic scan to achieve an optimal full width half maximum of 10 Hz. Total acquisition time for the ^1^H-MRS sequence was 5 min and 28 s.

The analyses of the spectral dataset were performed with the manufacturer-supplied software package program of the MR system (Sun, Advantage windows ADW4.5, General Electric, Milwaukee, WI, USA). Voxels were repositioned in the left and right LN. Each individual VOI was composed of four voxels (1 × 1 × 1 mm). The final measurement value of the individual VOI was the average value of the measurement value of four voxels. Each spectrum was evaluated for the peak area of Cho at 3.22 ppm, Cr at 3.03 ppm, and NAA at 2.02 ppm (showed in Figure 1B). A trained radiologist who was blinded to the diagnosis of each participant carried out voxel placement for spectroscopy. The values of the NAA/Cr and Cho/Cr ratios were used for analyses.

### Statistical Analysis

Data analysis was performed using SPSS 24.0 (SPSS Inc., Chicago, IL, USA). Two-tailed significance level was set at *p* < 0.05. All indicators (demographics, clinical data, executive function, metabolic data, and the levels of serum copper) were assessed for normal distribution using the Kolmogorov-Smirnov test and using the Levene's test for equality of error variances.

When comparing group differences in terms of demographic and clinical data, the Student *t* test was used if distribution was normal and the Mann-Whitney *U* test was used if distribution was skewed. The chi-square test was performed to compare the differences in sex between MDD and controls.

Second, Student *t* test and the Mann-Whitney *U* test was used to test the differences in biochemical metabolism ratios, serum copper levels and executive function. A Bonferroni *post hoc* test was applied to correct for multiple comparisons, with the threshold for significance was set to *p* < 0.004 (adjusted α = 0.004, 0.05/12).

Third, we performed *Pearson* correlation analyses to establish altered serum copper levels, brain biochemical metabolite ratios, and executive performance for patients with MDD.

Finally, multiple linear regression analyses were performed in R version 3.3.1. and adopted to model the relationship between serum copper levels, brain neurometabolite ratios, and executive performance. We set the significance levels at *p* < 0.05.

## Results

### Demographics and Cognition of Unmedicated MDD Patients and Healthy Controls

Demographic, clinical data, and executive function performance for MDD patients and healthy controls were listed in Table 1. There were no significant differences in sex, and age between the two groups (*p* > 0.05). Patients with MDD had a significantly lower score of DSCS, the CC number of WCST and SVFT, and significantly higher in the TE, PE number of WCST and TMT-B time compared with controls. Those differences of DSST, PE number of WCST, TMT-B time and SVFT were still exhibit after Bonferroni correction at *p* < 0.004.

**Table 1:** Comparison of clinical and demographic and EF performance between patients with MDD and healthy controls.

	**MDD**	**HCs**	**χ^2^/*t*/*Z***	***p***
Number of subjects	41	50	—	—
Sex (male/female)	15/26	23/27	0.821	0.365^a^
Age (year)	28.07 ± 10.11	24.70 ± 7.11	−0.980	0.327^b^
Number of episodes	1.66 ± 1.05	NA	NA	NA
Education(years)	12.24 ± 3.34	15.40 ± 1.99	−4.417	<0.001^b^^***^
Duration of illness (month)	46.08 ± 56.39	NA	NA	NA
24-item HDRS score	24.15 ± 6.48	0–7	NA	NA
Y-MRS score	0–6	0–6	NA	NA
DSST	55.85 ± 12.76	69.02 ± 10.41	−5.216	<0.001^b^^***^
CC,WCST	5.73 ± 1.58	6.52 ± 1.04	−248	0.025^b^^*^
TE,WCST	10.24 ± 9.55	5.18 ± 4.28	−2.686	0.007^b^^**^
PE,WCST	6.15 ± 7.38	2.18 ± 2.59	−3.220	0.001^b^^**^
NPE, WCST	4.17 ± 3.44	3.00 ± 2.38	−1.693	0.090^b^
TMT-B Time	62.57 ± 28.01	46.34 ± 14.09	−3.606	<0.001^b^^***^
SVFT	19.93 ± 5.87	23.18 ± 4.34	−3.036	0.003^c^^**^

*MDD, major depressive disorder; HCs, healthy controls; 24-item HDRS, 24 item Hamilton Depression Rating Scale; YMRS, Young Manic Rating Scale; WCST, Wisconsin Card Sorting Test-modified; CC, Categories Completed, WCST; TE, Total Errors WCST; PE, Perseverative Errors WCST; NPE, Nonperseverative Errors WCST; DSST, Digit Symbol Substitution Test; SVFT, Semantic Verbal Fluency testing*.

a *χ^2^ test*;

b *Mann-Whitney U test*;

c *t-test*.

*p < 0.05, **p < 0.01, ***p < 0.001.

### Serum Copper Levels and Neurometabolism in MDD Patients and Healthy Controls

The MDD group showed significantly higher serum copper levels compared to HCs (*t* = 3.745, *p* < 0.001) (Table 2). Patients with MDD had a significantly lower NAA/Cr ratio in the left LN compared to controls (*z* = −2.808, *p* = 0.006), but this results failed to exhibit any significant difference in the Bonferroni corrections at *p* < 0.004.

**Table 2:** Comparison of serum copper levels and neurometabolic characteristics between patients with MDD and healthy controls.

	**MDD**	**HCs**	**χ^2^/*t*/*Z***	***p***
Number of subjects	41	41	—	—
Sex (male/female)	15/26	20/21	1.246	0.264^a^
Age (year)	28.07 ± 10.11	26.49 ± 7.88	−0.260	0.795^b^
Number of episodes	1.66 ± 1.05	NA	NA	NA
Duration of illness (month)	46.08 ± 56.39	NA	NA	NA
24-item HDRS score	24.15 ± 6.48	0–7	NA	NA
Y-MRS score	0–6	0–6	NA	NA
Right LN Cho/Cr	0.77 ± 0.29	0.89 ± 0.63	−1.415	0.157^b^
Left LN Cho/Cr	0.76 ± 0.17	0.89 ± 0.57	−0.343	0.731^b^
Right LN NAA/Cr	1.66 ± 0.0.44	1.69 ± 0.25	−1.651	0.099^b^
Left LN NAA/Cr	1.52 ± 0.22	1.69 ± 0.32	−2.808	0.006^b^^**^
serum copper levels	16.90 ± 4.72	13.69 ± 2.79	3.745	<0.001^c^^***^

*MDD, major depressive disorder; HCs, healthy controls; 24-item HDRS, 24 item Hamilton Depression Rating Scale; LN, lenticular nucleus of basal ganglia; YMRS, Young Manic Rating Scale*.

a *χ^2^ test*;

b *Mann-Whitney U test*;

c *t-test*.

**p < 0.01, ***p < 0.001.

### Correlation Between Abnormal Serum Copper, Cognition and Neurometabolism in MDD

In the MDD group, serum copper levels were found to significantly negative associated with CC number of WCST (*r* = −0.408, *p* = 0.008), and significantly positive associated with the TE and PE number of WCST (*r* = 0.356, *p* = 0.023; *r* = −0.356, *p* = 0.022) (Table 3). In addition, the NAA/Cr ratios of left LN were found to significantly negative associated with VFS (*r* = −0.401, *p* = 0.009), as well as negative associated with serum copper levels (*r* = −0.365, *p* = 0.019).

**Table 3:** Correlation between abnormal serum copper, cognition and neurometabolism in MDD.

	**Left LN NAA/Cr**	**Serum copper levels**
DSST	−0.113	0.227
CC,WCST	0.177	−0.408**
TE,WCST	−0.221	0.356*
PE,WCST	−0.209	0.356*
TMT-B Time	0.041	−0.071
SVFT	−0.401**	0.263
Left LN NAA/Cr	NA	−0.365*
serum copper levels	−0.365*	NA

*p < 0.05, **p < 0.01.

*WCST, Wisconsin Card Sorting Test-modified; CC, Categories Completed; WCST; TE, Total Errors WCST; PE, Perseverative Errors WCST; NPE, Nonperseverative Errors WCST; DSST, Digit Symbol Substitution Test; SVFT, Semantic Verbal Fluency testing; LN, lenticular nucleus of basal ganglia*.

### Interaction of Abnormal Serum Copper Levels and Neurometabolic Ratios With Cognitive Function in Patients With MDD

Multiple regression analyses demonstrated that the Cu^2+^ × NAA interactions in the left LN was independent predictors for the SVFT (*t* = −0.736, *p* = 0.04) and WCST CC (*t* = −1.482, *p* = 0.02) in patients with MDD.

## Discussion

The purpose of our study has been to explore the underlying copper-brain interactions in patients with MDD compared with healthy controls. Ultimately, we found that (1) significant associations between abnormal serum copper levels and executive function deficits in patients with MDD, showing a significantly negative correlation of serum copper levels to CC number of WCST, along with a significantly positive correlation of copper to TE and PE number of WCST; (2) abnormal serum copper levels were also associated with neurometabolic disruption of patients with MDD, showing that copper levels had a significantly inverse association with NAA/Cr ratios in the left LN; and, (3) the interaction of abnormal copper levels and NAA/Cr neurometabolic disruption of the LN was independent predictors for the SVFT and WCST CC in patients with MDD.

Abnormal serum copper is a potential biomarker of MDD (13, 14). In our present study, we found significantly higher serum copper levels of patients with MDD when compared with healthy controls, findings consistent with previous studies (38, 39). The deposition of copper result in neuronal cell and gliocyte swelling and cystic degeneration, death, and loss, which induce persistent neurological deficits and cognitive dysfunction (40). In the present study, we found that patients with MDD had a significantly lower score of DSST, the CC number of WCST and Verbal fluency, and significantly higher in the TE, PE number of WCST and TMT-B time compared with controls. These results suggest that patients with MDD showed executive dysfunction, consisting with previous research findings (41). Correlation analysis shown that altered serum copper levels were found to significantly associated with CC, TE and PE number of WCST, suggesting a positive relationship between the higher serum copper levels and cognitive impairment risk. A meta-analysis demonstrated increased serum copper concentrations of AD patients when compared with healthy controls (42). Similarly, a previous study also reported that women with a high concentration of copper in plasma had poorer cognitive function than women with low plasma copper concentration (43). The alteration of Cu^2+^ homeostasis plays an essential role in the synthesis and functioning of BDNF (44). Copper regulates matrix metalloproteinases and tyrosine phosphatase activity, which promotes the maturation of pro-BDNF to BDNF (45, 46), and induces the oxidative stress damage (47). Moreover, a recent study indicated that the interaction between decreased BDNF and oxidative damage is involved in the disruption of executive function (48). In alignment with our findings, these results corroborates the notion that copper is essential for the synthesis of neurotransmitters and resultant impairment in cognitive function.

In our present study, we found MDD patients had a significantly lower NAA/Cr ratio in the left LN compared to controls, findings consistent with previous studies (30). NAA is synthesized in neuronal mitochondria and reflects the functional state of neurons. Reduced NAA levels may reflect a loss of neurons or may be a marker of neuronal damage and mitochondrial dysfunction (49, 50). Further, we found that the higher copper levels had a significantly inverse association with NAA/Cr ratios in the left LN of patients with MDD. Studies have suggested that the cuprizone (CPZ, a copper chelator) may damage oligodendrocytes by causing demyelinating insult and leading to the appearance of abnormal mental functions, such as schizophrenia symptoms (51). Rats on the CPZ diet showed decreased expression of mRNA transcripts encoding oligodendroglia proteins and displayed a specific deficit in the ability to shift between perceptual dimensions in the attentional set-shifting task, similar to WCST deficits observed in schizophrenia (52). And we did find an significantly negative association between decreased NAA/Cr ratios of left LN and executive dysfunction in patients with MDD. Although the associations cannot provide for causal pathophysiology, it has been suggested that abnormal serum copper levels may impair LN microstructural and eventually generate cognitive impairment.

Furthermore, we also found that the Cu × NAA interaction in the left LN was associated with poor executive performance in patients with MDD. Such changes may suggested that abnormal serum copper levels may impair LN microstructural and cause cognitive dysfunction. Here we are able to connect the neurometabolic abnormalities to altered serum copper levels through glutamate system. Firstly, the glutamatergic system also plays an important role in the pathophysiology of MDD (53). And the altered NAA may have a close relationship to and complex interaction with increased glutamate levels (54). Meanwhile, free copper excess alters their cortical glutamatergic neurotransmission (55). Recent studies have shown that copper plays an important role in the regulation of the NMDAR, the α-amino-3-hydroxy-5-methyl-4isoxazole-propionic acid receptor (AMPAR), synaptogenesis and learning and memory function (56, 57). In our recent study, we found that the NMDA receptor antagonist can suppress serum copper levels and improving symptoms of anhedonia and anxiety and the cognitive deficits associated with depression (58). Furthermore, copper is involved in oxidative stress processes and immunological system regulation (59). Depression is accompanied by the activation of oxidative stress and inflammatory reaction. And the increased inflammation in major depression may lead to increased glutamate in the basal ganglia in association with glial dysfunction (60), and thus impaired executive function (61). Taken together, these data indicate that the disruption of NAA/Cr neurometabolic of LN and serum copper levels is relevant to the pathophysiology of executive impairment in MDD patients.

## Limitations

We acknowledge that our study was limited in several ways. Namely, the sample size was relatively small and the participants were highly selected since the study was not a cross-sectional design. Furthermore, other factors may influence executive function: evidence also implicates childhood trauma and early life adversity as risk factors for cognitive deficits (62). Additionally, BDNF, oxidative stress or inflammatory and the levels of glutamate nor γ-aminobutyrate were not detected that might impact NAA levels over time, which could contribute to brain network and cognitive dysfunction. Further research incorporating biological and psychosocial effects, as well as longitudinal research may provide a more complete picture of the relationship between serum copper levels, neurometabolic changes and executive dysfunction.

## Conclusions

In conclusion, we conducted a correlation analysis on the relationship between serum copper, executive function, and neurometabolites of the LN in unmedicated patients with MDD. Our results indicate that patients with MDD show deficits in executive function and higher serum copper levels, in addition to a significantly negative correlation of serum copper levels and poorer executive performance. Patients with MDD also showed significantly lower NAA/Cr ratios in LN compared to healthy controls. Meanwhile, the abnormal serum copper levels were also associated with decreased NAA/Cr ratios in the left LN. Further, our results indicated that the interaction of abnormal copper levels and NAA/Cr neurometabolic disruption of the LN may impact executive dysfunction in patients with MDD. Despite the previously described limitations, we believe this may relevant to the pathophysiology of executive impairment in MDD patients.

## Data Availability Statement

The raw data supporting the conclusions of this article will be made available by the authors, without undue reservation.

## Ethics Statement

The studies involving human participants were reviewed and approved by the Ethics Committee of the First Affiliated Hospital of Jinan University, China. All subjects signed a written informed consent form after a full written and verbal explanation of the study. The patients/participants provided their written informed consent to participate in this study.

## Author Contributions

YJ, XL, SZ, and SL were involved in the design of this study and contributed to the writing of the manuscript. GC and FC performed the MR scans. GC, FC, and YW performed the MR data analyses. YJ and SZ were involved in diagnosing subjects. SL, SZ, and Yan Xia carried out the statistical analyses. YZ, SS, and HH were involved in clinical data collection. XL, SL, and SZ wrote the first draft of the manuscript. YJ provided critical revisions of the the manuscript. All authors contributed to the article and approved the submitted version.

## Conflict of Interest

The authors declare that the research was conducted in the absence of any commercial or financial relationships that could be construed as a potential conflict of interest.
